# Health Information Sourcing and Health Knowledge Quality: Repeated Cross-sectional Survey

**DOI:** 10.2196/39274

**Published:** 2022-09-28

**Authors:** Elena Korshakova, Jessecae K Marsh, Samantha Kleinberg

**Affiliations:** 1 Department of Computer Science Stevens Institute of Technology Hoboken, NJ United States; 2 Department of Psychology Lehigh University Bethlehem, PA United States

**Keywords:** health knowledge, health information seeking, information dissemination, COVID-19, online health information, public health, health literacy, social media, information quality, infodemiology

## Abstract

**Background:**

People’s health-related knowledge influences health outcomes, as this knowledge may influence whether individuals follow advice from their doctors or public health agencies. Yet, little attention has been paid to where people obtain health information and how these information sources relate to the quality of knowledge.

**Objective:**

We aim to discover what information sources people use to learn about health conditions, how these sources relate to the quality of their health knowledge, and how both the number of information sources and health knowledge change over time.

**Methods:**

We surveyed 200 different individuals at 12 time points from March through September 2020. At each time point, we elicited participants’ knowledge about causes, risk factors, and preventative interventions for 8 viral (Ebola, common cold, COVID-19, Zika) and nonviral (food allergies, amyotrophic lateral sclerosis [ALS], strep throat, stroke) illnesses. Participants were further asked how they learned about each illness and to rate how much they trust various sources of health information.

**Results:**

We found that participants used different information sources to obtain health information about common illnesses (food allergies, strep throat, stroke) compared to emerging illnesses (Ebola, common cold, COVID-19, Zika). Participants relied mainly on news media, government agencies, and social media for information about emerging illnesses, while learning about common illnesses from family, friends, and medical professionals. Participants relied on social media for information about COVID-19, with their knowledge accuracy of COVID-19 declining over the course of the pandemic. The number of information sources participants used was positively correlated with health knowledge quality, though there was no relationship with the specific source types consulted.

**Conclusions:**

Building on prior work on health information seeking and factors affecting health knowledge, we now find that people systematically consult different types of information sources by illness type and that the number of information sources people use affects the quality of individuals’ health knowledge. Interventions to disseminate health information may need to be targeted to where individuals are likely to seek out information, and these information sources differ systematically by illness type.

## Introduction

The quality and credibility of information sources people use to find health-related information play a major role in their health outcomes because this information contributes to people’s health knowledge. Research has shown that false information can lead to poor treatment choices in many disorders [1], increased worry [2], and, more recently, reduced intentions to receive COVID-19 vaccinations [3]. Although extremely abundant, the content of online health information sources remains mostly unregulated [4], and people often use their own judgment to evaluate these information sources [5,6]. Internet sources are frequently used, though patients also rely on health care professionals and use other sources, such as family, friends, and newspapers, to supplement their information [7]. Further, as awareness of a novel illness grows, people’s knowledge and information sources may also change. During the early stages of the COVID-19 pandemic, there was an increase in online information seeking about COVID-19 [8], with a decrease in searches about other health conditions [9]. People searched for information about COVID-19 and its symptoms and visited social media in response to news stories, but these effects were short lived [10,11]. Given the variety in the type and quality of information available, it is crucial to understand the relationship between where people gather their health information and the quality of their health knowledge, and how this may change over time.

We aim to determine (1) what information sources people trust and use to gather health information, (2) the relationship between information sources used and health knowledge quality, and (3) how people’s sources of health information change during an evolving situation, the COVID-19 pandemic, and how this relates to the quality of their health knowledge. A better understanding of the link between health information sources and health knowledge quality could allow health care professionals to redirect patients to reliable information sources, and facilitate shared decision-making by providing insight into patients’ current understanding. Exploring all of this during a pandemic allows us to understand how the use of information sources may change as knowledge of a disease accrues.

### Background

We first review research on where people obtain health information, before examining people’s health knowledge and finally discuss the link between information sources and knowledge.

#### Health Information Sourcing

As individuals have access to increasing amounts of health information outside of direct interaction with their doctors, understanding where people obtain this information has become critically important. Models of health information–seeking behavior (HISB) aim to capture the process by which individuals engage in information seeking and how this relates to their health outcomes. Johnson’s [12,13] comprehensive model of information seeking (CMIS) has 3 components: antecedents, meaning factors (eg, beliefs) that influence information seeking; information carrier characteristics (eg, content, sources); and the resulting actions. Although this model has been applied widely, it was originally developed based on information seeking around breast cancer. However, other work has found that people may avoid information about cancer and genetic screening for it [14], especially when they believe themselves at risk [15], suggesting that information seeking related to cancer may differ from other conditions. Another limitation is not accounting for individuals seeking out health information for other people in their lives [16].

Much work around HISB has focused on the information sources individuals use [17] and prefer [18], with particular emphasis on online sources [19] due to their prevalence [20]. Search engines and social media are the primary sources of health information for the US population [4]. Many adults consult the internet before consulting their doctor [21], particularly when they are dissatisfied with their medical care [22]. The most common health search queries fall into 4 main groups: (1) general understanding of a health condition or diagnosis; (2) treatment options, including procedures or medications; (3) information about health professionals and institutions, such as hospitals and pharmacies; and (4) diet and lifestyle information for chronic concerns [23,24]. Among the major search engines, Google was found to provide the most useful and relevant information for health-related searches [25], while Twitter and YouTube were the most popular social media platforms for health information searches [1,4].

One advantage over static information sources (eg, academic and government websites) is that social media platforms allow people to interact and share information with others. However, as experts do not curate these information sources, their quality is unknown, and their use could negatively affect health decisions and behavior [1]. Wikipedia, a free, open source online encyclopedia, is a middle ground, as the information is not vetted by experts but the community attempts to maintain quality and accuracy. Wikipedia is viewed as a reliable source of information by 64% of Americans regardless of the topic choice [26]. Yet, among Wikipedia’s more than 14,000 entries on health topics, only 4% were confirmed as high quality [27]. As people rarely check the quality of online health information, they may be unaware of being exposed to low-quality or false information.

Most prior studies on health information seeking focused on online sources, so less is known about how and when people seek out and use offline information sources, such as medical professionals, family members, or news media [7]. Jacobs et al [28] found that most people go to the internet first, with few people seeking information from family, friends, and coworkers. However, that study looked at health information seeking in general, rather than for specific conditions, so it is still an open question whether people’s behavior may differ for commonly experienced conditions, such as a cold, compared to emerging diseases, such as Zika virus. Zhang [29] interviewed individuals about specific health incidents, finding factors influencing how people select information sources, but again did not examine differences across conditions or how sources relate to knowledge.

Beyond a preference to look online, where people choose to obtain health information depends on the health topic, information availability, privacy concerns, quality of the information people expect to find, and health literacy [4,30]. When concerned about privacy and searching for information about conditions where there may be social stigma, people avoid social media and instead use search engines, journals, and books to learn about their health conditions [4]. Some studies have found a correlation between sociodemographic characteristics and patterns of online health information seeking [31,32]. For example, LaValley et al [33] found that younger people tend to use commercial websites (eg, vitamin suppliers who also provide health information to engage people in e-commerce). In contrast, older people tend to use academic websites. Other work has found that overall older adults are less likely to trust online sources [34] and are less successful at using them [35]. Additionally, men use fewer information sources than women and are more concerned about accuracy, comprehensiveness, and ease of access, while women pay more attention to interpretability and ease of understanding [25]. Religious and charitable organizations, which provide access to free or low-cost health care, are common health information sources for vulnerable populations, including ethnic minorities, people with limited English proficiency, rural residents, and immigrants [36]. Overall, prior work has explored the sources used for health information seeking either in specific demographic groups for general information search or for one specific disorder at a time. However, there has been no research that explores within 1 study how information seeking differs across disease types, thus allowing for extrapolating across types of disease. We instead aim to understand whether there are systematic disease-based differences in where people go for health information.

#### Health Knowledge

The information people gather about health and illness adds to their health knowledge, meaning their beliefs about factors influencing health, causes of disease, and ways to treat and prevent illness [37]. Health knowledge influences how and whether people follow health guidelines [38-40] and when inaccurate can lead to a wide range of adverse health outcomes and delayed medical care [41,42]. Incorrect health knowledge can contribute to choosing a suboptimal treatment and consequently worsen people’s conditions, which is particularly risky for acute diseases [43,44].

Models of health information seeking, such as the CMIS, posit that beliefs influence how people seek out health information, but do not examine the process of belief formation. Many of these models are influenced by and incorporate aspects of the Health Belief Model (HBM), which represents the main factors affecting people’s health-related behaviors [45]. An alternative model that is more relevant to our study is the Common-Sense Model of Self-Regulation (CSM) [46,47]. In this model, the individual is thought of as (1) actively solving a problem by seeking information, testing hypotheses about their health, and relating their experiences to information they receive; (2) having an illness representation that guides their behaviors; and (3) having their own beliefs that may differ from others’ and from medical consensus. This model suggests that experiences, such as an individual’s own experiences or those of family or friends, change illness representations. Thus, over the course of a pandemic where people initially have no personal experience and then gain experience as well as exposure to media, the CSM predicts beliefs will change. The beliefs that have been studied most systematically are those surrounding medication, finding that beliefs about the necessity of medication and reduced concerns are associated with adherence to treatment [48]. However, this work has not been linked to individuals’ information-seeking behavior, so it is not known whether the quality of health knowledge is related to the information sources people consult. To close this gap, we aim to conduct an exploratory analysis of how people’s knowledge of causes, risk factors, and preventative measures for different illnesses relates to the information sources they use to search for health information.

#### How Health Information Sourcing Affects Health Knowledge

Although the importance of people’s health knowledge is well established, relatively less is known about how that knowledge is influenced by the specific information sources people use. Kealey and Berkman [49] found a correlation between the health information sources participants use and their mental models of cancer. Participants who learned about cancer from the local news had greater ambiguity about cancer prevention, while people who searched for cancer-related information on the internet and in newspapers had less ambiguity [49]. Relying on lay information sources of health information, such as friends and family, was associated with having a higher likelihood of incorrect beliefs about skin cancer [50]. According to both the HBM and the CSM, these beliefs influence behavior. For example, for mental health conditions, people most often report getting information from personal experiences with diagnosed individuals, and these experiences change perceptions of a host of factors, including the causes of disease and treatment preferences (for a review, see [51]). However, the relationship is not necessarily only in 1 direction, as behaviors can influence information seeking, such as anxiety moderating the relationship between care use and information seeking [52].

Information sources themselves have also been linked to behavior, with individuals using print media and interpersonal sources as information sources being more likely to engage in health behaviors [53]. However, far fewer works have examined how information can influence beliefs. Smokers who sought out health information were more likely to intend to quit, but researchers did not find beliefs to mediate this relationship [54].

Although prior work suggests that information matters to whether people understand how to prevent disease, research into the relationship between specific information sources and health knowledge has been limited. Further, we must better understand where people obtain information and how it influences their beliefs across different types of conditions rather than for health in general or only specific concerns. A better understanding of this relationship may aid medical professionals and policymakers who deliver health information and inform the general population. Thus, in this study, we aim to provide a deeper understanding of health information sourcing, trust in those information sources, and implications for health knowledge by investigating the relationship between where people obtain health information and what they know about various illnesses.

## Methods

### Materials

We selected 8 illnesses, including both viral (Ebola, common cold, COVID-19, Zika) and nonviral (food allergies, amyotrophic lateral sclerosis [ALS], strep throat, stroke) illnesses. These were chosen to include a mix of severities across viral and nonviral illnesses and within nonviral illnesses to have an even split of chronic (food allergies, ALS) and acute (strep throat, stroke) conditions. Given the time frame during which the study was run, we expected COVID-19 to be on many participants’ minds. Including other viral illnesses allowed for comparison to other similarly caused health conditions as a baseline against which COVID-19–related responses could be compared.

For each illness, participants first saw 1 screen with the following 3 questions with [ITEM] filled in with the name of the corresponding illness:

- What do you think leads to [ITEM]? (cause question)
- What makes people more or less likely to develop [ITEM]? (risk factor question)
- How can [ITEM] be prevented? (prevention question)

On the next screen, participants were asked to reflect on where they got this information, with the following prompt (information source question):

Now we’d like you to list all the places where you think you learned any or all of the information you listed about [ITEM]. If you can’t remember where you learned something, describe where you think you would have gone to find this information.

Some examples of sources include a particular newspaper or magazine, a specific website, personal experience, a medical professional, or from a family member or friend. This is not exhaustive and there may be other sources as well. Please be as specific as possible.

All responses were free text. After completing these questions for all 8 illnesses, participants rated the trustworthiness of 16 sources of health information, ranging from 1 (not at all trustworthy) to 7 (extremely trustworthy). Participants were also able to indicate whether they were unable to judge a source’s trustworthiness. The 16 unique information sources were chosen from places individuals were a priori thought to use for health information (search engines, doctors, WebMD, government health organizations, Wikipedia, public health campaigns, TV news, news websites or newspapers, family, friends), along with information sources mentioned by participants during pilot testing (social media, health and fitness magazines, YouTube, Reddit, medical journals, personal experience). We ran the survey using Qualtrics.

### Ethical Considerations

The data were collected with Institutional Review Board (IRB) approval from the Stevens Institute of Technology (IRB protocol #2018-003). Before beginning the study, participants read a consent document informing them about the purpose of the study and noting the voluntary nature of participation. Participants provided consent by clicking a response button.

### Procedure

Participants were recruited over 12 time points using Prolific and were compensated US $4.50 for participation. We restricted the age range as prior work has found that older adults exhibit significantly different information-seeking behavior online [33,55] and different patterns of trust in such sources [34]. Payment was lower during the first time point (US $3), but we found the study duration to be longer than expected (mean 29.77 minutes, SD 17.21 across all time points) and increased payment accordingly to maintain the target hourly rate. A total of 2350 (97.92%) of 2400 participants remained in analysis, as 4 (0.17%) did not complete the study and 46 (1.91%) were excluded: 17 (37%) responses were random sentences unrelated to the question, 17 (37%) were copied from the internet or the instructions, 7 (15%) were duplicates, 4 (9%) participants reported being aged <18 years or >65 years, and 1 (2%) response was written in Polish.

After consenting to the study, all participants completed the 4 questions (cause, risk factor, prevention, information source) for each illness, with the order of illnesses randomized, followed by the trust ratings for the various predetermined information sources. Following this, participants completed a demographic questionnaire, which, in addition to age, gender, and education level, asked about their state of residence and COVID-19–related restrictions where the participants lived. The survey was run every other week for 20 weeks starting March 31, 2020 (10 time points), and then again 4 weeks later and 2 weeks after that (September 22, 2020) for a total of 12 time points. This frequency was chosen to be often enough to capture changes during the beginning phase of the pandemic. The 2 final time points were chosen to be 1 week before and 2 weeks after Labor Day. These dates coincided with a time in the United States when people often travel and the start of the school year, both events predicted to lead to a surge in COVID-19 cases. Individuals were only allowed to participate in 1 time point.

### Participants

We recruited 200 participants per time point across the 12 time points (2400 total) using Prolific. All participants were US residents aged 18-64 years. Multimedia Appendix 1 includes detailed demographic information about the 2350 participants in our analysis. Across all 12 time points, our sample included 1081 (46%) women, 1222 (52% men), and 47 (2%) who identified in other ways. Participants were mainly younger adults, with 775 (33%) being 18-24, 869 (37%) being 25-34, 400 (17%) being 35-44, 212 (9%) being 45-54, and 94 (4%) being 55-64 years old.

### Data Analysis

All data on causes, risk factors, and preventive measures were coded independently by 2 coders, with disagreements discussed and resolved either by the coders or by a third party. Information sources were coded at a fine level of granularity (eg, biology class) and then mapped to higher-level categories (eg, education). Responses in which participants indicated that they did not know (I do not know [IDK]) were excluded from analysis at the item level (eg, response to ALS risk factors excluded for a participant coded as IDK, while ALS causes and prevention remained in analysis). For each condition, we determined whether individual responses were correct using the websites of government agencies responsible for international public health, namely the Centers for Disease Control and Prevention (CDC), the World Health Organization, the American Stroke Association, and the Asthma and Allergy Foundation of America.

To evaluate health knowledge quality about causes, risk factors, and preventive measures, we assigned 0 to incorrect responses and 1 to correct ones. See Multimedia Appendices 2 and 3 for a list of all codes used and what responses were considered correct for each illness, Multimedia Appendix 4 for where the correct answers were drawn from, and Multimedia Appendix 5 for an example of how 1 participant’s responses were coded. Responses to the cause and risk factor prompts were similar, with participants often listing the same factors for both prompts, so these were combined for analysis. We calculated knowledge precision and knowledge depth (recall) as follows. Knowledge precision is the number of correct unique responses for a participant divided by the total number of unique responses by that participant. For cause and risk factor categories, as long as the response was accurate for either cause or risk factors, it was considered correct. We then averaged precision across cause/risk factor and prevention to yield 1 precision score for each individual for each illness. We calculated knowledge depth by dividing a participant’s number of correct responses for an illness and category by the number of codes considered correct for that illness and category. Thus, if the CDC and other sources list A and B as causes or risk factors of COVID-19 and Amy lists only B, Amy’s precision is 1.0 and recall/knowledge depth is 0.5. Together, these measures indicate what fraction of a participant’s responses are correct (knowledge precision) and the extent of their knowledge about a condition (knowledge depth).

As our study was exploratory, we conducted a series of analyses to answer key open questions about health information sourcing. We first examined where people obtain information (using prevalence of listed sources) and how sources used differ across illnesses (using a factor analysis). Our factor analysis was run with maximum likelihood estimation using direct oblimin rotation. We used the Scree test and retained all factors with an eigenvalue of 1 or greater. We then examined how much people trust various information sources in general, and then compared average trust in sources an individual used to sources they did not use, tested with the Kolmogorov-Smirnov test. The third key facet we examined is quality of knowledge. We used the coded responses to compare knowledge quality across illnesses and then examined whether knowledge quality is correlated with the number of sources consulted. Finally, we examined how knowledge quality about COVID-19 and the understanding of local restrictions related to the pandemic changed over time.

## Results

### Where Do People Obtain Their Health Information?

We begin with the analysis of the first time point (March 31, 2020) before examining how results changed over time. To determine where people obtain their health information, we report the percentage of individuals mentioning each high-level information source type (eg, news, rather than CNN) for each illness (Table 1). See Multimedia Appendix 6 for the full list of high-level information source types.

**Table 1:** Top 10 information sources participants used to obtain health information by illness (N=200).

Information source	Food allergies, n (%)	ALS^a^, n (%)	Common cold, n (%)	COVID-19, n (%)	Ebola, n (%)	Strep throat, n (%)	Stroke, n (%)	Zika, n (%)	Total^b^, n (%)
News	16 (8)	20 (10)	16 (8)	66 (33)	70 (35)	6 (3)	10 (5)	66 (33)	36 (18)
Family	24 (12)	10 (5)	30 (15)	12 (6)	8 (4)	30 (15)	36 (18)	8 (4)	20 (10)
Medical professional	20 (10)	4 (2)	24 (12)	6 (3)	2 (1)	38 (19)	18 (9)	6 (3)	14 (7)
Government agency	2 (1)	2 (1)	2 (1)	28 (14)	12 (6)	2 (1)	2 (1)	12 (6)	12 (6)
Social media	4 (2)	16 (8)	4 (2)	18 (9)	14 (7)	2 (1)	4 (2)	10 (5)	12 (6)
Friend	24 (12)	6 (3)	12 (6)	10 (5)	6 (3)	14 (7)	8 (4)	6 (3)	10 (5)
Education	18 (9)	10 (5)	22 (11)	2 (1)	8 (4)	12 (6)	22 (11)	4 (2)	10 (5)
Internet	12 (6)	10 (5)	10 (5)	6 (3)	10 (5)	10 (5)	6 (3)	8 (4)	8 (4)
Website	8 (4)	2 (1)	6 (3)	4 (2)	6 (3)	14 (7)	8 (4)	6 (3)	6 (3)
Personal experience	8 (4)	0	12 (6)	0	0	22 (11)	2 (1)	0	6 (3)

^a^ALS: amyotrophic lateral sclerosis.

^b^Total represents the percentage of participants mentioning each information source when combining all illnesses for analysis of the first time point (March 31, 2020). This value was used for sorting information sources in the table.

We conducted exploratory factor analysis to determine whether there are subgroups or patterns in the information sources used. For all illnesses (N=8), we input the number of mentions for each high-level information source (N=55), leading to an 8×55 matrix where each cell is a count of mentions for that source type for that illness, across all participants. We found that illnesses clustered into 2 distinct groups (Tables 2 and 3), with 4 illnesses loaded (≥.9) on the first factor and 3 other illnesses loaded (>.9) on the second factor. One group, which we refer to as “emerging illnesses,” included newly appearing infectious diseases significantly impacting public health (Zika, COVID-19, and Ebola), while the other, “common illnesses,” included those illnesses that historically have been frequent among the general population (stroke, strep throat, food allergies, and common cold). The most frequently mentioned sources for emerging illnesses were the news (n=68, 34% mentions), government agencies (n=18, 9%), and social media (n=14, 7%). For common illnesses, the most frequently mentioned sources were family (n=30, 15%), medical professionals (n=26, 13%), and friends (n=14, 7%). ALS differed significantly from these 2 groups, reflecting that people learned about it in idiosyncratic ways. Additionally, many participants reported not knowing anything about ALS. When participants did report an information source, it was often related to social media campaigns (eg, ice bucket challenge) or information about celebrities, so this is less likely to reflect intentional information seeking, and ALS was excluded from further analysis.

**Table 2:** Rotated component matrix.

	Factor 1	Factor 2
Common cold	.952	N/A^a^
Strep throat	.941	N/A
Stroke	.915	N/A
Food allergies	.904	.323
Ebola	N/A	.976
Zika	N/A	.972
COVID-19	N/A	.942
ALS^b^	.348	.680

^a^N/A: not applicable.

^b^ALS: amyotrophic lateral sclerosis.

**Table 3:** Correlation of information sources used between illnesses.

	ALS^a^	Common cold	COVID-19	Ebola	Strep throat	Stroke	Zika
Food allergies	.21	.9	.22	.24	.76	.76	.23
ALS	N/A^b^	.2	.61	.74	–.15	.18	.69
Common cold	N/A	N/A	.14	.18	.84	.87	.17
COVID-19	N/A	N/A	N/A	.94	–.12	.03	.94
Ebola	N/A	N/A	N/A	N/A	–.11	.03	.99
Strep throat	N/A	N/A	N/A	N/A	N/A	.66	–.11
Stroke	N/A	N/A	N/A	N/A	N/A	N/A	.06

^a^ALS: amyotrophic lateral sclerosis.

^b^N/A: not applicable.

### Do People Use the Information Sources They Find Trustworthy?

**Figure 1:**
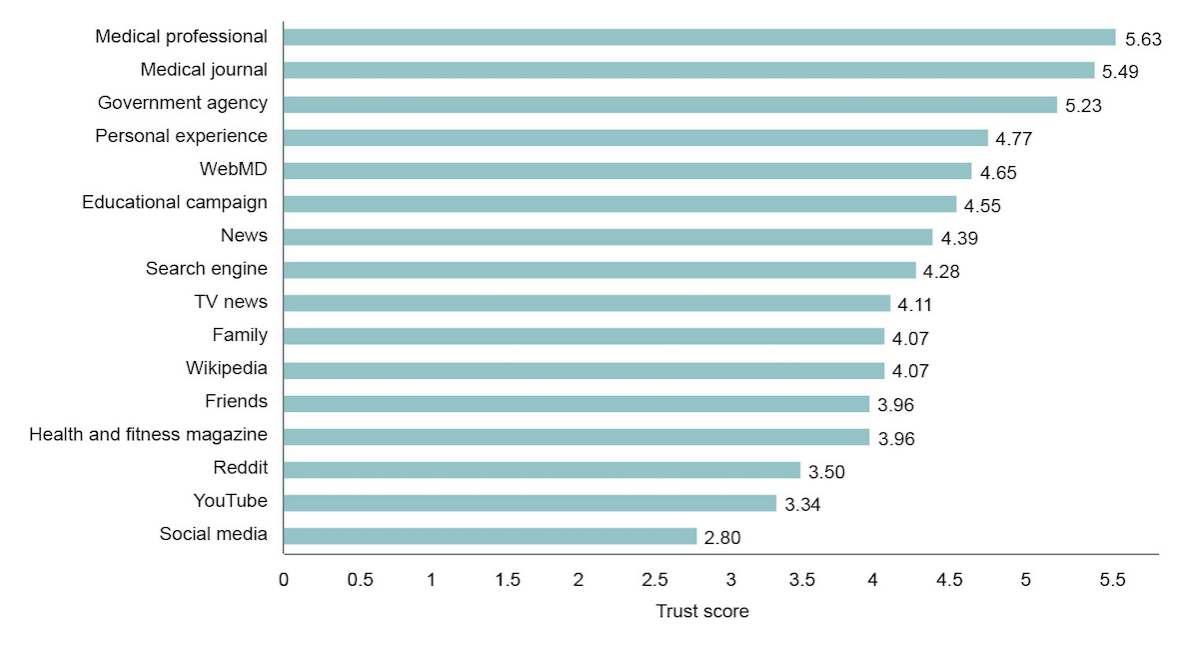
Average trust score by category for time point 1 (March 31, 2020).

We now examine how participants’ perception of the trustworthiness of information sources relates to those they consult. First, we calculated average trust in the 16 information sources participants rated at the first time point. Social media received the lowest rating (mean 2.8, SD 1.21), followed by YouTube (mean 3.34, SD 1.23) and Reddit (mean 3.50, SD 1.41), while medical professional (mean 5.63, SD 1.03), medical journal (mean 5.49, SD 1.96), and government agency (mean 5.23, SD 1.41) were rated as the most trustworthy (Figure 1).

To calculate people’s trust in the information sources they reported using, we mapped the sources each individual used to learn about each illness to the 16 information sources they rated (eg, school nurse was assigned the rating given for medical professional). We then performed the Kolmogorov-Smirnov test to compare the average trust scores for information sources each participant used to the average trust scores for sources they did not use for common and emerging illnesses. The ratings were averaged for unique information sources participants used for each group of illnesses (common and emerging) and separately for sources participants did not use. We found that trust scores were significantly higher for sources participants used to learn about both common illnesses (D_198_=.26, *P*<.001; used mean 4.32, SD 1.11; not used mean 3.97, SD 1.23) and emerging illnesses (D_198_=.39, *P*<.001; used mean 4.52, SD 1.09; not used mean 4.03, SD 1.17), meaning people considered the information sources they used more trustworthy than those they did not use. Thus, even if social media is not widely considered a reliable source of health information, compared to individuals who do not use it, individuals who use it believe it is more reliable.

### Are Information Sources Correlated With Knowledge Quality?

To evaluate participants’ knowledge quality and understand its relationship to information sourcing, we averaged knowledge precision and knowledge depth separately by participant within each individual illness group (eg, we averaged knowledge precision for COVID-19, Zika, and Ebola to represent emerging illnesses; detailed data by illness are shown in Multimedia Appendix 7). As the data are not normally distributed (see Multimedia Appendices 8-11), we performed the Kolmogorov-Smirnov test to compare both knowledge precision and knowledge depth between common and emerging illnesses for statistical significance. We found that knowledge precision for emerging illnesses was significantly higher than for common illnesses (D_198_=.27, *P*=.003). To examine the extent of participants’ knowledge, we now turn to analysis of knowledge depth (Table 4). We found again that participants had significantly higher scores for emerging (mean 0.67, SD 0.23) versus common (mean 0.51, SD 0.27) illnesses (D_198_=.19, *P*<.001). Finally, we performed Spearman rank correlation to test the relationship between the mean number of information sources participants used and the quality of their knowledge. We found that the number of information sources participants reported using was weakly positively correlated with knowledge precision for both common (r_198_=.15, *P*<.001) and emerging (r_198_=.12, *P*<.001) illnesses. We observed a stronger correlation between knowledge depth and the number of information sources reported for both common (r_198_=.31, *P*<.001) and emerging (r_198_=.29, *P*<.001) illnesses.

**Table 4:** Knowledge precision and knowledge depth for common and emerging illnesses.

Emerging illnesses		Common illnesses	
Knowledge precision, mean (SD)	Knowledge depth, mean (SD)	Knowledge precision, mean (SD)	Knowledge depth, mean (SD)
0.53 (0.19)	0.67 (0.23)	0.45 (0.18)	0.51 (0.27)

### How Do Knowledge Quality and the Number of Information Sources Change Over Time for COVID-19?

**Figure 2:**
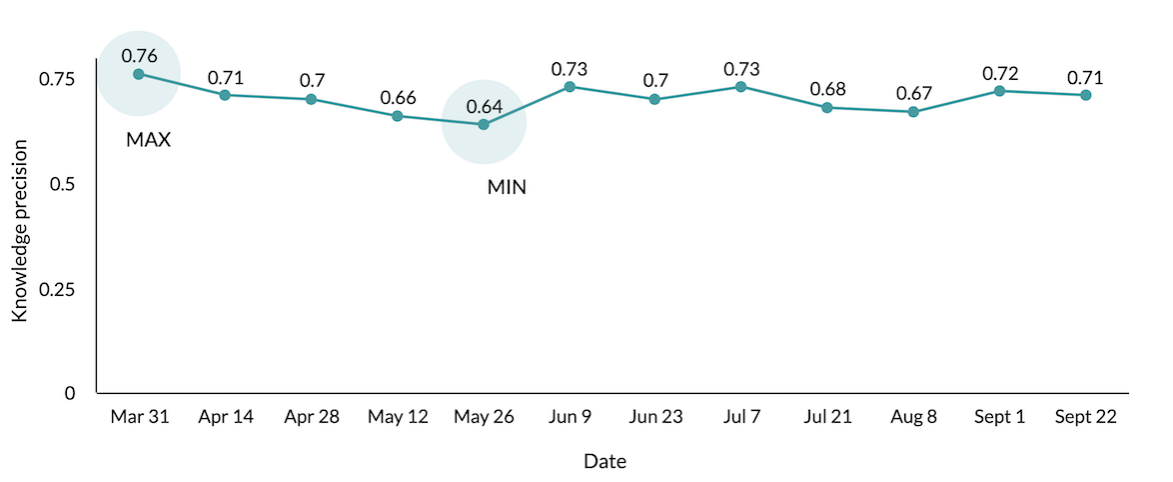
Knowledge precision (combining causes, risk factors, and prevention) about COVID-19 across all 12 time points.

Our second key analysis examined how information sources and trust in sources changed over time from March 31 to September 22, 2020, for COVID-19. We ran a linear trend analysis using the number of information sources participants listed and their trust scores across all 12 time points. We did not find significant changes over time in the number of information sources used (F_1,184_=8.23, mean-square error [MSE]=0.16, *P*=.37) or average trust scores across all 16 information sources (F_1,184_=12.76, MSE=0.22, *P*=.45), nor did we find a change in trust in any individual source (all *P*>.09). However, we did observe significant changes in knowledge quality using linear trend analysis. The highest knowledge precision score for COVID-19 was found at the start of data collection, on March 31, 2020 (mean 0.76, SD 0.19), when knowledge depth was also high (mean 0.72, SD 0.12). Both knowledge precision and knowledge depth significantly decreased (knowledge precision: F_1,184_=5.31, MSE=0.25, *P*=.03; knowledge depth: F_1,184_=3.02, MSE=0.18, *P*<.001) by the last time point, on September 22, 2020 (knowledge precision: mean 0.71, SD 0.22; knowledge depth: mean 0.69, SD 0.18), as shown in Figure 2. Additionally, we measured the effect size, representing the drop in accuracy from one time point to the next using the Cohen d score. We found that the effect size between time points varied from small to medium (d=0.32-0.49). These findings suggest that people were reporting more incorrect information, in addition to correct information, as time went on. Notably, we did not find a significant difference over time for either emerging or common illnesses (excluding COVID-19) for the number of sources used (common: F_1,184_=6.65, MSE=0.11, *P*=.19; emerging: F_1,184_=7.89, MSE=0.14, *P*=.09), trust in sources (common: F_1,184_=9.86, MSE=0.15, *P*=.49; emerging: F_1,184_=14.21, MSE=0.09, *P*=.77), knowledge precision (common: F_1,184_=10.31, MSE=0.13, *P*=.08; emerging: F_1,184_=13.64, MSE=0.11, *P*=.35) or knowledge depth (common: F_1,184_=11.73, MSE=0.14, *P*=.21; emerging: F_1,184_=8.67, MSE=0.08, *P*=.45).

Similarly, we examined how knowledge about COVID-19–related restrictions changed over time to find out how aware participants were of rapidly changing health guidelines. We collapsed all states for analysis (after finding no significant difference in scores between regions of the United States, nor based on state-level vaccination rates, which could indicate differences in COVID-19–related beliefs). Linear trend analysis showed a decline in both knowledge precision (F_1,184_=12.91, MSE=0.19, *P*=.003) and knowledge depth (F_1,184_=8.004, MSE=0.26, *P*<.001) of COVID-19–related restrictions. Additionally, we examined the types of incorrect responses by comparing how often people thought there were more restrictions versus fewer. To compare these scores, we calculated the number of incorrect responses by participant within these 2 categories and compared them for statistical significance using the Kolmogorov-Smirnov test. Incorrect responses were mainly due to participants being unaware of restrictions in their state and believing there were fewer restrictions than were actually imposed (D_1649_=0.23, *P*<.001).

## Discussion

### Principal Findings

In this study, we aimed to learn what information sources people use to learn about health conditions, how these sources relate to the quality of their health knowledge, and how both information source choices and health knowledge change over time. First, we found that people systematically rely on different types of sources, depending on whether they are obtaining information about common illnesses (food allergies, strep throat, stroke) or emerging illnesses (Ebola, common cold, COVID-19, Zika), with individuals relying on the news, government agencies, and social media for gathering information about emerging illnesses, while seeking information from family, friends, and medical professionals for common illnesses. Second, we found that the number of sources people use is positively correlated with the quality of health knowledge. Interestingly, participants had a better understanding of emerging illnesses than common ones, which may be explained by the fact that participants were more likely to use lay information sources, such as friends and family, to learn about common illnesses, suggesting those information sources are more likely to be incomplete or incorrect.

Although participants overall did distinguish between the trustworthiness of different information sources, they rated sources they used as more trustworthy than those they did not use. For example, although social media received low trust scores overall, compared to individuals who do not use it as a source of health information, those who do find it more trustworthy. Finally, throughout 6 months of the early stage of the COVID-19 pandemic, we found that knowledge quality overall declined, including knowledge of causes, risk factors, and preventative interventions, along with knowledge of local COVID-19–related restrictions. We did not find any change over time for the other conditions, which suggests that despite our cross-sectional approach, changes in knowledge over time were specific to COVID-19 and not due to population differences. This decrease in knowledge may be in part because over the course of the pandemic, the number of different, nuanced, restrictions grew. As the complexity in restrictions grew, it appears our participants were less able to keep track of what was in place in their state. Future work should explore how the complexity of public health restrictions relates to the knowledge of those restrictions and how these findings can be used to develop guidelines on information dissemination for emerging illnesses.

### Comparison With Prior Work

Our study can be understood in the context of the CSM [47], whereby illness representations, which include knowledge of causes and control (eg, preventive strategies, treatment), guide behavior. Building on this framework, we now find that people’s knowledge of both differs systematically between common and emerging illnesses as do their information sources. Although the link between beliefs and specific source choices has not been examined, prior work has framed information search as a hypothesis-testing process, suggesting that beliefs may guide what people search for online [56]. It remains for future work to determine whether information quality (eg, use of lay and interpersonal sources) drives reduced knowledge quality for common illnesses or alternatively whether personal factors independently drive both knowledge and source selection. The CSM also provides a framework for interpreting our findings on COVID-19. In addition to the growing complexity of COVID-19 knowledge and restrictions over time (eg, just stay home vs guidelines on masking, isolation after infection, and ventilation), people may also perceive the health threat differently over time as they gain personal experience or find that it is increasingly well understood. This in turn may influence their information sources or information-seeking behavior. Future work during emerging pandemics is needed to examine the relationship between information sources, beliefs, and perceived risk.

We also built on prior work on health information seeking by identifying systematic differences across illnesses. Previous studies have focused primarily on the use of online health information sources, such as social media and websites [1,2,4,31], and people’s knowledge about specific illnesses [5,21,22] rather than finding patterns in illness groups. Instead of focusing on specific information sources, we used open-ended questions collected across a range of times, enabling us to learn both where people obtain their information and how this may change. Our findings regarding the most common health information sources are aligned with prior work investigating health information–seeking strategies [1,2,4]; however, prior studies did not investigate search patterns by illness group and their correlation with health knowledge. Differences in the sources of information that people use for emerging and common illnesses may be explained by the nature of the illness. Common illnesses, such as food allergies, the common cold, strep throat, and stroke, may be learned about from family members over the course of a lifetime as they affect individuals or those they know. When a new disorder emerges, individuals do not have personal experience and they rely on news media and government agencies, both of which disseminate information about novel illnesses [57]. This difference provides an opportunity to extend our findings to other illnesses that may be categorized as either common or emerging diseases and to develop effective information delivery strategies.

We further found that although overall, people rate medical professionals and government sources of information as more trustworthy than lay sources (eg, social media, friends), compared to individuals who do not use lay information sources for obtaining health information, people who choose to do so rate those sources as more trustworthy. This has 2 key implications. First, there is a need to help individuals access reliable information and to evaluate the quality of information they receive. People may be at risk of making wrong decisions because of misconceptions regarding their health and lack of awareness of the quality of the health information they use. This could contribute to inappropriate health interventions, misinterpretation of new information, and poor health outcomes. Second, knowing where individuals are looking for information during a health emergency, such as a pandemic, versus when they have a common condition means that information can be selectively targeted to where individuals will look for it. For example, Bautista et al [58] proposed having health professionals correct misinformation on social media. Our results provide further support for this. Wider health communication campaigns on social media can focus on using medical professionals to provide reliable information about emerging illnesses, such as the ongoing coronavirus pandemic. Knowing that participants commonly use social media even though they recognize it as less trustworthy can be leveraged by government agencies to more effectively deliver information, such as about vaccination, and potentially improve health knowledge.

### Limitations

Since our data were collected from Prolific and only from US residents, our study may not generalize to other populations. Thus, future research may be needed to investigate the relationship between information sources and health knowledge quality in geographically diverse samples. The nature of the illnesses studied is also such that we cannot learn in real time where people obtained information (eg, when they first heard about strep throat), so there may be bias due to relying on participants’ memory and surveying different participants over time. As we did not ask participants whether they had any of the listed conditions, it is possible that personal experience may be a moderator of information sources or knowledge quality. Using multiple categories of illnesses allows us to see that participants are not simply listing the sources they used for the most recent emerging illness (COVID-19), but we cannot know whether an individual’s specific information sources or knowledge quality are changing. Our analyses were mainly exploratory, though conducting many analyses could introduce α errors. Further, although multiple coders independently coded each free-text response, this qualitative analysis process could potentially introduce bias. Finally, we solicited ratings of trust for 16 categories of information sources, but this did not cover more infrequently mentioned source types and combined categories of sources (eg, family, without distinguishing between people in a caregiving role or by closeness of the relationship).

Other key avenues for future research include examining trust at a more granular level (eg, the specific sources each participant listed), changes in belief and information seeking at the individual level (eg, to discover whether an individual may shift from social media to family for information as an illness becomes endemic in a population), how using an information source for an emerging disease may change information source use for previously known diseases, and how these findings can be used to deliver information more effectively. Although we know what misconceptions people may have and where they are seeking information, future work is needed to translate this into effective message strategies.

### Conclusion

Our study investigated what information sources people use to learn health information and how these sources are related to individuals’ knowledge. The results of our study indicate that health information sources and knowledge quality vary by illness type. We identified 2 groups of illnesses according to information sources people consult to find health information: common illnesses (food allergies, strep throat, stroke) that are mainly learned from family, friends, and medical professionals and emerging illnesses (Ebola, common cold, COVID-19, Zika) that people learn about from news media, government agencies, and social media. We also found that people tend to trust information sources they use even when most people consider them low quality (eg, social media). Our findings suggest a need for targeted interventions via information sources people are likely to use for different illness types. In the future, we aim to extend this study by investigating how incorrect knowledge affects the way people use information and make health-related decisions.

## Appendix

Multimedia Appendix 1 Percentage distribution of participants by demographic.

Multimedia Appendix 2 List of codes for causes and risk factors and correctness by illness.

Multimedia Appendix 3 List of codes for preventative measures and correctness by illness.

Multimedia Appendix 4 List of sources for assigning the correctness of participants’ responses.

Multimedia Appendix 5 Coding protocol sample.

Multimedia Appendix 6 List of all high-level information sources used for categorizing participant responses.

Multimedia Appendix 7 Knowledge precision and depth across the illnesses on March 31 (timepoint 1).

Multimedia Appendix 8 Knowledge precision distribution for emerging illnesses.

Multimedia Appendix 9 Knowledge precision distribution for common illnesses.

Multimedia Appendix 10 Knowledge depth distribution for emerging illnesses.

Multimedia Appendix 11 Knowledge depth distribution for common illnesses.

## Abbreviations

ALS: amyotrophic lateral sclerosis
CDC: Centers for Disease Control and Prevention
CMIS: comprehensive model of information seeking
CSM: Common-Sense Model of Self-Regulation
HBM: Health Belief Model
HISB: health information–seeking behavior
IDK: I do not know
MSE: mean-square error

## References

[1] Madathil KC, Rivera-Rodriguez AJ, Greenstein JS, Gramopadhye AK (2015). Healthcare information on YouTube: a systematic review. Health Inform J.

[2] Liu PL (2020). COVID-19 information seeking on digital media and preventive behaviors: the mediation role of worry. Cyberpsychol Behav Soc Netw.

[3] Loomba S, de Figueiredo A, Piatek SJ, de Graaf K, Larson HJ (2021). Measuring the impact of COVID-19 vaccine misinformation on vaccination intent in the UK and USA. Nat Hum Behav.

[4] De Choudhury M, Morris MR, White RW (2014). Seeking and sharing health information online: comparing search engines and social media.

[5] Sillence E, Briggs P, Harris PR, Fishwick L (2007). How do patients evaluate and make use of online health information?. Soc Sci Med.

[6] Sun Y, Zhang Y, Gwizdka J, Trace CB (2019). Consumer evaluation of the quality of online health information: systematic literature review of relevant criteria and indicators. J Med Internet Res.

[7] Cutilli CC (2010). Seeking health information: what sources do your patients use?. Orthop Nurs.

[8] Jokic-Begic N, Lauri Korajlija A, Mikac U (2020). Cyberchondria in the age of COVID-19. PLoS One.

[9] Mangono T, Smittenaar P, Caplan Y, Huang VS, Sutermaster S, Kemp H, Sgaier SK (2021). Information-seeking patterns during the COVID-19 pandemic across the United States: longitudinal analysis of google trends data. J Med Internet Res.

[10] Bento AI, Nguyen T, Wing C, Lozano-Rojas F, Ahn Y, Simon K (2020). Evidence from internet search data shows information-seeking responses to news of local COVID-19 cases. Proc Natl Acad Sci.

[11] Gozzi N, Tizzani M, Starnini M, Ciulla F, Paolotti D, Panisson A, Perra N (2020). Collective response to media coverage of the COVID-19 pandemic on Reddit and Wikipedia: mixed-methods analysis. J Med Internet Res.

[12] Johnson J, Meischke H (1993). A comprehensive model of cancer-related information seeking applied to magazines. Human Commun Res.

[13] Johnson JD (1997). Cancer-Related Information Seeking.

[14] Case DO, Andrews JE, Johnson JD, Allard SL (2005). Avoiding versus seeking: the relationship of information seeking to avoidance, blunting, coping, dissonance, and related concepts. J Med Libr Assoc.

[15] Brashers DE, Goldsmith DJ, Hsieh E (2002). Information seeking and avoiding in health contexts. Human Commun Res.

[16] Reifegerste D, Blech S, Dechant P (2020). Understanding information seeking about the health of others: applying the comprehensive model of information seeking to proxy online health information seeking. J Health Commun.

[17] Anker AE, Reinhart AM, Feeley TH (2011). Health information seeking: a review of measures and methods. Patient Educ Couns.

[18] Zimmerman MS, Shaw G (2020). Health information seeking behaviour: a concept analysis. Health Info Libr J.

[19] Jia X, Pang Y, Liu LS (2021). Online Health Information Seeking Behavior: A Systematic Review. Healthcare.

[20] Cline RJ, Haynes KM (2001). Consumer health information seeking on the internet: the state of the art. Health Educ Res.

[21] Swoboda CM, Van Hulle JM, McAlearney AS, Huerta TR (2018). Odds of talking to healthcare providers as the initial source of healthcare information: updated cross-sectional results from the Health Information National Trends Survey (HINTS). BMC Fam Pract.

[22] Tustin N (2010). The role of patient satisfaction in online health information seeking. J Health Commun.

[23] Wang L, Wang J, Wang M, Li Y, Liang Y, Xu D (2012). Using Internet search engines to obtain medical information: a comparative study. J Med Internet Res.

[24] Bach RL, Wenz A (2020). Studying health-related internet and mobile device use using web logs and smartphone records. PLoS One.

[25] Rowley J, Johnson F, Sbaffi L (2015). Gender as an influencer of online health information-seeking and evaluation behavior. J Assoc Inf Sci Technol.

[26] Javanmardi S, Ganjisaffar Y, Lopes C, Baldi P (2009). User contribution and trust in Wikipedia.

[27] Laurent MR, Vickers TJ (2009). Seeking health information online: does Wikipedia matter?. J Am Med Inform Assoc.

[28] Jacobs W, Amuta AO, Jeon KC (2017). Health information seeking in the digital age: an analysis of health information seeking behavior among US adults. Cogent Soc Sci.

[29] Zhang Y (2014). Beyond quality and accessibility: source selection in consumer health information searching. J Assoc Inf Sci Technol.

[30] Diviani N, Zanini C, Jaks R, Brach M, Gemperli A, Rubinelli S (2020). Information seeking behavior and perceived health literacy of family caregivers of persons living with a chronic condition. The case of spinal cord injury in Switzerland. Patient Educ Couns.

[31] Ali SH, Foreman J, Tozan Y, Capasso A, Jones AM, DiClemente RJ (2020). Trends and predictors of COVID-19 information sources and their relationship with knowledge and beliefs related to the pandemic: nationwide cross-sectional study. JMIR Public Health Surveill.

[32] Perez SL, Kravitz RL, Bell RA, Chan MS, Paterniti DA (2016). Characterizing internet health information seeking strategies by socioeconomic status: a mixed methods approach. BMC Med Inform Decis Mak.

[33] LaValley SA, Kiviniemi MT, Gage-Bouchard EA (2017). Where people look for online health information. Health Info Libr J.

[34] Miller LMS, Bell RA (2012). Online health information seeking: the influence of age, information trustworthiness, and search challenges. J Aging Health.

[35] Agree EM, King AC, Castro CM, Wiley A, Borzekowski DLG (2015). "It's got to be on this page": age and cognitive style in a study of online health information seeking. J Med Internet Res.

[36] Wheldon CW, Carroll KT, Moser RP (2020). Trust in health information sources among underserved and vulnerable populations in the U.S. J Health Care Poor Underserved.

[37] Reynolds R, Dennis S, Hasan I, Slewa J, Chen W, Tian D, Bobba S, Zwar N (2018). A systematic review of chronic disease management interventions in primary care. BMC Fam Pract.

[38] Marsh JK, Ungson ND, Packer DJ (2021). Bring out your experts: the relationship between perceived expert causal understanding and pandemic behaviors. J Exp Psychol Appl.

[39] Brewer NT, Chapman GB, Gibbons FX, Gerrard M, McCaul KD, Weinstein ND (2007). Meta-analysis of the relationship between risk perception and health behavior: the example of vaccination. Health Psychol.

[40] Bruine de Bruin W, Bennett D (2020). Relationships between initial COVID-19 risk perceptions and protective health behaviors: a national survey. Am J Prev Med.

[41] Livi S, Zeri F, Baroni R (2017). Health beliefs affect the correct replacement of daily disposable contact lenses: predicting compliance with the Health Belief Model and the Theory of Planned Behaviour. Cont Lens Anterior Eye.

[42] Vitriol JA, Marsh JK (2021). A pandemic of misbelief: how beliefs promote or undermine COVID-19 mitigation. Front Polit Sci.

[43] Prasanna SS, Korner-Bitensky N, Ahmed S (2013). Why do people delay accessing health care for knee osteoarthritis? Exploring beliefs of health professionals and lay people. Physiother Can.

[44] Jurkowski JM, Maniccia DM, Spicer DA, Dennison BA (2010). Impact of a multimedia campaign to increase intention to call 9-1-1 for stroke symptoms, upstate New York, 2006-2007. Prev Chronic Dis.

[45] Jones CL, Jensen JD, Scherr CL, Brown NR, Christy K, Weaver J (2015). The Health Belief Model as an explanatory framework in communication research: exploring parallel, serial, and moderated mediation. Health Commun.

[46] Leventhal H, Phillips LA, Burns E (2016). The Common-Sense Model of Self-Regulation (CSM): a dynamic framework for understanding illness self-management. J Behav Med.

[47] Leventhal H, Brissette I, Leventhal EA, Cameron LD, Leventhal H (2003). The common-sense model of self-regulation of health and illness. The Self-Regulation of Health and Illness Behaviour.

[48] Horne R, Chapman SCE, Parham R, Freemantle N, Forbes A, Cooper V (2013). Understanding patients' adherence-related beliefs about medicines prescribed for long-term conditions: a meta-analytic review of the Necessity-Concerns Framework. PLoS One.

[49] Kealey E, Berkman CS (2010). The relationship between health information sources and mental models of cancer: findings from the 2005 Health Information National Trends Survey. J Health Commun.

[50] Ford BM, Kaphingst KA (2009). Lay interpersonal sources for health information related to beliefs about the modifiability of cancer risk. Cancer Causes Control.

[51] Jorm AF (2000). Mental health literacy. Public knowledge and beliefs about mental disorders. Br J Psychiatry.

[52] Eastin MS, Guinsler NM (2006). Worried and wired: effects of health anxiety on information-seeking and health care utilization behaviors. Cyberpsychol Behav.

[53] Redmond N, Baer HJ, Clark CR, Lipsitz S, Hicks LS (2010). Sources of health information related to preventive health behaviors in a national study. Am J Prev Med.

[54] Upadhyay S, Lord J, Gakh M (2019). Health-information seeking and intention to quit smoking: do health beliefs have a mediating role?. Tob Use Insights.

[55] Sharit J, Hernández Mario A, Czaja SJ, Pirolli P (2008). Investigating the roles of knowledge and cognitive abilities in older adult information seeking on the web. ACM Trans Comput Hum Interact.

[56] Keselman A, Browne AC, Kaufman DR (2008). Consumer health information seeking as hypothesis testing. J Am Med Inform Assoc.

[57] Green MH (2020). Emerging diseases, re‐emerging histories. Centaurus.

[58] Bautista JR, Zhang Y, Gwizdka J (2021). Healthcare professionals' acts of correcting health misinformation on social media. Int J Med Inform.

