# Supplementary material for: Health Information Sourcing and Health Knowledge Quality: Repeated Cross-sectional Survey
Source: JMIR Form Res. 2022 Sep 28;6(9):e39274. doi: 10.2196/39274 (PMC9557754; doi:10.2196/39274)
Supplement: Multimedia Appendix 1 [file formative_v6i9e39274_app1.docx]

| **Timepoint** | **1** | **2** | **3** | **4** | **5** | **6** | **7** | **8** | **9** | **10** | **11** | **12** | **Total** |
| --- | --- | --- | --- | --- | --- | --- | --- | --- | --- | --- | --- | --- | --- |
| Age range | | | | | | | | | | | | | |
| 18–24 | 0.23 | 0.32 | 0.42 | 0.38 | 0.33 | 0.43 | 0.31 | 0.27 | 0.36 | 0.2 | 0.37 | 0.33 | 0.33 |
| 25–34 | 0.34 | 0.4 | 0.37 | 0.31 | 0.38 | 0.36 | 0.36 | 0.37 | 0.36 | 0.48 | 0.39 | 0.36 | 0.37 |
| 35–44 | 0.21 | 0.13 | 0.15 | 0.2 | 0.17 | 0.09 | 0.21 | 0.21 | 0.18 | 0.2 | 0.13 | 0.19 | 0.17 |
| 45–54 | 0.12 | 0.11 | 0.04 | 0.07 | 0.11 | 0.1 | 0.09 | 0.1 | 0.07 | 0.06 | 0.07 | 0.08 | 0.09 |
| 55–64 | 0.1 | 0.04 | 0.02 | 0.04 | 0.01 | 0.02 | 0.03 | 0.05 | 0.03 | 0.06 | 0.04 | 0.04 | 0.04 |
| Gender | | | | | | | | | | | | | |
| Male | 0.44 | 0.42 | 0.48 | 0.52 | 0.5 | 0.43 | 0.44 | 0.47 | 0.36 | 0.56 | 0.42 | 0.46 | 0.46 |
| Female | 0.55 | 0.55 | 0.49 | 0.47 | 0.48 | 0.55 | 0.54 | 0.49 | 0.63 | 0.43 | 0.57 | 0.53 | 0.52 |
| Other | 0.01 | 0.03 | 0.01 | 0.01 | 0.01 | 0.02 | 0.01 | 0.04 | 0.01 | 0 | 0.01 | 0.01 | 0.01 |
| Prefer not to say | 0 | 0 | 0.02 | 0 | 0.01 | 0 | 0.01 | 0 | 0 | 0.01 | 0 | 0 | 0 |
| Race | | | | | | | | | | | | | |
| American Indian or Alaska Native | 0 | 0.01 | 0 | 0.02 | 0 | 0.04 | 0.01 | 0 | 0.03 | 0.02 | 0.01 | 0.03 | 0.01 |
| Asian | 0.08 | 0.08 | 0.1 | 0.1 | 0.1 | 0.11 | 0.07 | 0.09 | 0.08 | 0.04 | 0.1 | 0.04 | 0.08 |
| Black or African American | 0.04 | 0.06 | 0.06 | 0.12 | 0.08 | 0.09 | 0.08 | 0.11 | 0.08 | 0.12 | 0.12 | 0.06 | 0.09 |
| Hispanic or Latino | 0.05 | 0.08 | 0.06 | 0.08 | 0.08 | 0.08 | 0.06 | 0.07 | 0.06 | 0.04 | 0.04 | 0.04 | 0.06 |
| White | 0.74 | 0.63 | 0.62 | 0.62 | 0.63 | 0.56 | 0.62 | 0.6 | 0.63 | 0.61 | 0.63 | 0.64 | 0.63 |
| Multiple races indicated | 0.08 | 0.03 | 0.05 | 0.03 | 0.06 | 0.05 | 0.04 | 0.03 | 0.04 | 0.02 | 0.08 | 0.03 | 0.05 |
| Other | 0.01 | 0.1 | 0.11 | 0.03 | 0.03 | 0.07 | 0.11 | 0.1 | 0.08 | 0.14 | 0.01 | 0.16 | 0.08 |
| Prefer not to say | 0 | 0.01 | 0 | 0 | 0.02 | 0 | 0.01 | 0 | 0 | 0.01 | 0.01 | 0 | 0.01 |
| Complete education | | | | | | | | | | | | | |
| None completed | 0.01 | 0 | 0.02 | 0.02 | 0.02 | 0.02 | 0.07 | 0.03 | 0 | 0.06 | 0 | 0.03 | 0.02 |
| High school | 0.33 | 0.31 | 0.33 | 0.35 | 0.36 | 0.33 | 0.37 | 0.33 | 0.33 | 0.29 | 0.32 | 0.3 | 0.33 |
| Bachelor’s or Associate degree | 0.48 | 0.54 | 0.48 | 0.46 | 0.47 | 0.44 | 0.41 | 0.46 | 0.45 | 0.42 | 0.48 | 0.45 | 0.46 |
| Graduate degree | 0.18 | 0.11 | 0.14 | 0.17 | 0.15 | 0.18 | 0.11 | 0.16 | 0.18 | 0.21 | 0.18 | 0.21 | 0.17 |
| Other | 0 | 0.04 | 0.03 | 0 | 0 | 0.03 | 0.04 | 0.02 | 0.04 | 0.02 | 0.02 | 0.01 | 0.02 |
