# Supplementary material for: Health Information Sourcing and Health Knowledge Quality: Repeated Cross-sectional Survey
Source: JMIR Form Res. 2022 Sep 28;6(9):e39274. doi: 10.2196/39274 (PMC9557754; doi:10.2196/39274)
Supplement: Multimedia Appendix 2 [file formative_v6i9e39274_app2.docx]

| **Code** | **Code description** | **COVID** | **Cold** | **Ebola** | **Zika** | **ALS** | **Strep** | **Stroke** | **Allergies** |
| --- | --- | --- | --- | --- | --- | --- | --- | --- | --- |
| Age | Old age | 1 | 1 | 0 | 0 | 1 | 1 | 1 | 0 |
| Air | Air transmission, airborne | 1 | 1 | 0 | 0 | 0 | 0 | 0 | 1 |
| Allergy | Allergy | 0 | 0 | 0 | 0 | 0 | 0 | 0 | 0 |
| Animal | Any type of animal bite, not insect | 0 | 0 | 1 | 0 | 0 | 0 | 0 | 1 |
| AreaDis | Living in an area or visiting a specific region or area with mention that disease is prevalent there | 1 | 0 | 1 | 1 | 0 | 0 | 0 | 0 |
| AreaGen | Living in an area or visiting a specific region or area with no mention that disease is prevalent there | 0 | 0 | 0 | 0 | 0 | 0 | 0 | 0 |
| Bacteria | Bacteria, bacterial infection | 0 | 0 | 0 | 0 | 0 | 1 | 0 | 0 |
| BFlow | Blood flow issues | 0 | 0 | 0 | 0 | 0 | 0 | 1 | 0 |
| China | Originated in / blamed China | 0 | 0 | 0 | 0 | 0 | 0 | 0 | 0 |
| Cholesterol | High cholesterol | 0 | 0 | 0 | 0 | 0 | 0 | 1 | 0 |
| Congenital | Born with it | 0 | 0 | 0 | 1 | 0 | 0 | 0 | 0 |
| Contact | Contact with another person | 1 | 1 | 1 | 0 | 0 | 1 | 0 | 0 |
| Demo | Any demographic variables like race, ethnicity, gender (but not age) | 0 | 0 | 0 | 0 | 1 | 0 | 0 | 0 |
| Diet | Diet, food you eat, lack of good food | 0 | 0 | 0 | 0 | 0 | 0 | 1 | 1 |
| Drinking | Drinking | 0 | 0 | 0 | 0 | 0 | 0 | 1 | 0 |
| EarlyLife | Lack of exposure early in life, too much exposure early in life | 0 | 0 | 0 | 0 | 0 | 0 | 0 | 0 |
| Environment | Environment, living conditions | 0 | 0 | 0 | 0 | 0 | 0 | 0 | 0 |
| Exercise | Lack of exercise | 0 | 0 | 0 | 0 | 0 | 0 | 1 | 0 |
| Exposure | General exposure to the disease without mention of a specific person or area (to rule out Contact or Area) | 0 | 0 | 0 | 0 | 0 | 0 | 0 | 0 |
| Family | Parent has it, family history of some sort (that does not specifically mention genes) | 0 | 0 | 0 | 0 | 0 | 0 | 0 | 0 |
| Fluids | Any type of body fluid contact | 1 | 1 | 1 | 0 | 0 | 1 | 0 | 0 |
| Gene | Genetics, genes, some type of specifically genetic predisposition | 0 | 0 | 0 | 0 | 1 | 0 | 1 | 1 |
| Germs | General description of germ contact (not specifying bacteria or virus, just saying germs) | 0 | 0 | 0 | 0 | 0 | 0 | 0 | 0 |
| Hand | Lack of handwashing | 1 | 0 | 0 | 0 | 0 | 0 | 0 | 0 |
| Health | Poor health or overall well-being in a general, unspecified sense | 0 | 0 | 0 | 0 | 0 | 0 | 0 | 0 |
| Healthcare | Not going to your doctor, not getting treated by a doctor | 0 | 0 | 0 | 0 | 0 | 0 | 1 | 0 |
| Heart | Heart disease, heart conditions | 1 | 0 | 0 | 0 | 0 | 0 | 1 | 0 |
| Immune | Compromised immune system | 1 | 0 | 0 | 0 | 0 | 0 | 0 | 1 |
| Infection | Infection (not specified what type) | 0 | 0 | 0 | 0 | 0 | 0 | 0 | 0 |
| Insect | Any type of insect bite | 0 | 0 | 0 | 1 | 0 | 0 | 0 | 1 |
| Luck | Random chance, bad luck | 0 | 0 | 0 | 0 | 0 | 0 | 0 | 0 |
| Neuro | Neurological deficit, brain trauma, death of motor neurons | 1 | 0 | 0 | 0 | 0 | 0 | 0 | 0 |
| Plants | Pollen / Plants | 0 | 0 | 0 | 0 | 0 | 0 | 0 | 1 |
| Prexisting | Other prexisting conditions | 1 | 0 | 0 | 0 | 0 | 0 | 1 | 0 |
| Prevent | Not taking preventative measures, not following up on warning signs, not taking care of self, something generic about not doing something ahead of time. | 0 | 0 | 0 | 0 | 0 | 0 | 1 | 0 |
| Protect | Not wearing some type of protective clothing, spray | 1 | 0 | 0 | 1 | 0 | 0 | 0 | 0 |
| Sex | Sexual contact | 0 | 0 | 1 | 1 | 0 | 0 | 0 | 0 |
| Sleep | Not getting enough sleep | 0 | 0 | 0 | 0 | 0 | 0 | 0 | 0 |
| Smoking | Smoking of any things | 1 | 0 | 0 | 0 | 0 | 0 | 1 | 0 |
| SocDist | Not social distancing | 1 | 0 | 0 | 0 | 0 | 0 | 0 | 0 |
| Stress | High amounts of stress | 0 | 0 | 0 | 0 | 0 | 0 | 0 | 0 |
| Surface | Contaminated surfaces | 1 | 1 | 0 | 0 | 0 | 0 | 0 | 0 |
| Travel | Mention of traveling without talking about where. If talks generically about going to a place (e.g., travel to USA), then it should be AreaGen. If says travel to place where disease is, then should be AreaDis. | 0 | 0 | 0 | 0 | 0 | 0 | 0 | 0 |
| Unclean | Being unclean, dirty | 0 | 0 | 0 | 0 | 0 | 0 | 0 | 0 |
| Vaccines | Triggered by getting a vaccine | 0 | 0 | 0 | 0 | 0 | 0 | 0 | 0 |
| Varied | Not one thing leads to it, different factors, range of severity, different ways of transmission | - | - | - | - | - | - | - | - |
| Virus | Virus, viral infection | 1 | 1 | 1 | 1 | 0 | 1 | 0 | 0 |
| Weather | Weather | 0 | 0 | 0 | 0 | 0 | 0 | 0 | 1 |
| Weight | Being overweight | 1 | 0 | 0 | 0 | 0 | 0 | 1 | 0 |
| IDK | Just said didn't know | - | - | - | - | - | - | - | - |
| Other | Other | - | - | - | - | - | - | - | - |
