# Supplementary material for: Health Information Sourcing and Health Knowledge Quality: Repeated Cross-sectional Survey
Source: JMIR Form Res. 2022 Sep 28;6(9):e39274. doi: 10.2196/39274 (PMC9557754; doi:10.2196/39274)
Supplement: Multimedia Appendix 3 [file formative_v6i9e39274_app3.docx]

| **Code** | **Code description** | **COVID** | **Cold** | **Ebola** | **Zika** | **ALS** | **Strep** | **Stroke** | **Allergies** |
| --- | --- | --- | --- | --- | --- | --- | --- | --- | --- |
| Age | Reference to young or old age | 0 | 0 | 0 | 0 | 0 | 0 | 0 | 0 |
| Animal | Killing / removing animals that spread the disease | 0 | 0 | 1 | 0 | 0 | 0 | 0 | 1 |
| AreaAway | Staying away from a Region/area | 1 | 0 | 1 | 0 | 0 | 0 | 0 | 0 |
| Aware | Being aware that you are at risk, knowledge of risk factors, research on the disease, monitoring | 1 | 0 | 0 | 0 | 0 | 0 | 0 | 0 |
| BFlow | Good blood flow (pressure, clear arteries) | 0 | 0 | 0 | 0 | 0 | 0 | 1 | 0 |
| Cholesterol | Low cholesterol | 0 | 0 | 0 | 0 | 0 | 0 | 1 | 0 |
| Clean | Being clean | 0 | 0 | 0 | 0 | 0 | 0 | 0 | 0 |
| Diet | Healthy diet (something about eating in a healthy style; not just anything generic about eating) | 0 | 0 | 0 | 0 | 0 | 0 | 1 | 1 |
| Disinfect | Disinfecting surfaces | 1 | 1 | 0 | 0 | 0 | 0 | 0 | 0 |
| EarlyLife | Good exposure / environment early in life (including specific types of exposure early in life) | 0 | 0 | 0 | 0 | 0 | 0 | 0 | 0 |
| Exercise | Getting exercise | 0 | 0 | 0 | 0 | 0 | 0 | 1 | 0 |
| Family | Parent has it, family history of some sort (that does not specifically mention genes) | 0 | 0 | 0 | 0 | 0 | 0 | 0 | 0 |
| Gene | Genetics, genes, some type of specifically genetic predisposition | 0 | 0 | 0 | 0 | 0 | 0 | 0 | 0 |
| Hand | Handwashing | 1 | 1 | 0 | 0 | 0 | 1 | 0 | 0 |
| Healthcare | Medical check ups, doctor visits, testing | 1 | 0 | 0 | 0 | 0 | 0 | 1 | 0 |
| Healthy | Some reference to being overall healthy without any specficity | 0 | 0 | 0 | 0 | 0 | 0 | 0 | 0 |
| Hydration | Being well hydrated, staying hydrated | 0 | 0 | 0 | 0 | 0 | 0 | 0 | 0 |
| Immune | Strengthen immune system | 0 | 0 | 0 | 0 | 0 | 0 | 0 | 0 |
| Impossible | Can't be prevented | 0 | 0 | 0 | 0 | 0 | 0 | 0 | 0 |
| Meds | Some form of medication that is preventative (including vitamins) | 0 | 0 | 0 | 0 | 0 | 0 | 1 | 0 |
| Mental | Good mental health | 0 | 0 | 0 | 0 | 0 | 0 | 0 | 0 |
| NoContact | Staying away from contact with another person | 1 | 1 | 1 | 0 | 0 | 1 | 0 | 0 |
| Prevent | Preventative measures, injury prevention, warning signs, taking care of self, being careful, etc. | 1 | 0 | 0 | 0 | 0 | 0 | 0 | 0 |
| Protect | Wearing some type of protective clothing, spray | 1 | 0 | 0 | 1 | 0 | 0 | 0 | 0 |
| Screening | Some form of genetic or DNA screening before having children or while pregnant | 0 | 0 | 1 | 1 | 0 | 0 | 0 | 0 |
| Sleep | Getting good sleep | 0 | 0 | 0 | 0 | 0 | 0 | 0 | 0 |
| Smoking | Stop smoking or did not smoke | 0 | 0 | 0 | 0 | 0 | 0 | 1 | 0 |
| Substances | Any reference to use of alcohol or drugs (not cigarettes) | 0 | 0 | 0 | 0 | 0 | 0 | 1 | 0 |
| Stress | Lower stress levels | 0 | 0 | 0 | 0 | 0 | 0 | 0 | 0 |
| Vaccine | Vaccine | 1 | 0 | 1 | 0 | 0 | 0 | 0 | 0 |
| Weight | Healthy weight | 0 | 0 | 0 | 0 | 0 | 0 | 1 | 0 |
| IDK | Just said didn't know | - | - | - | - | - | - | - | - |
| Other | Other | - | - | - | - | - | - | - |  |
