# Supplementary material for: Health Information Sourcing and Health Knowledge Quality: Repeated Cross-sectional Survey
Source: JMIR Form Res. 2022 Sep 28;6(9):e39274. doi: 10.2196/39274 (PMC9557754; doi:10.2196/39274)
Supplement: Multimedia Appendix 4 [file formative_v6i9e39274_app4.docx]

| **Illness** | **Causes and risk factors** | **Prevention** |
| --- | --- | --- |
| Ebola | WHO, <https://www.who.int/health-topics/ebola#tab=tab_1> | WHO, <https://www.who.int/health-topics/ebola#tab=tab_3> |
| Common Cold | CDC, <https://www.cdc.gov/features/rhinoviruses/index.htm> | CDC,  <https://www.cdc.gov/features/rhinoviruses/index.htm> |
| COVID-19 | CDC, <https://www.cdc.gov/coronavirus/2019-nCoV/index.html> | CDC, <https://www.cdc.gov/coronavirus/2019-ncov/community/correction-detention/guidance-correctional-detention.html> |
| Zika | CDC, <https://www.cdc.gov/zika/about/index.html> | CDC, <https://www.cdc.gov/zika/prevention/index.html> |
| Food Allergies | AAFA, <https://www.aafa.org/food-allergies/> | AAFA, <https://www.aafa.org/prevent-allergies/> |
| ALS | CDC, <https://www.cdc.gov/als/WhatisAmyotrophiclateralsclerosis.html> | CDC, <https://www.cdc.gov/als/WhatisAmyotrophiclateralsclerosis.html> |
| Strep Throat | CDC, <https://www.cdc.gov/groupastrep/diseases-public/strep-throat.html> | CDC, <https://www.cdc.gov/groupastrep/diseases-public/strep-throat.html> |
| Stroke | ASA, https://www.heart.org/en/health-topics/high-blood-pressure/health-threats-from-high-blood-pressure/how-high-blood-pressure-can-lead-to-stroke | ASA, https://www.stroke.org/en/life-after-stroke/preventing-another-stroke |
