# Supplementary material for: Health Information Sourcing and Health Knowledge Quality: Repeated Cross-sectional Survey
Source: JMIR Form Res. 2022 Sep 28;6(9):e39274. doi: 10.2196/39274 (PMC9557754; doi:10.2196/39274)
Supplement: Multimedia Appendix 5 [file formative_v6i9e39274_app5.docx]

| **Survey question** | **Participant’s answer** | **Codes** | **Correctness** |
| --- | --- | --- | --- |
| What do you think leads to Ebola virus? | “Contact with someone else who has Ebola” | Contact | 1 |
| What do you think makes people more or less likely to develop Ebola virus? | “Weakened immune system” | Immune | 0 |
| How do you think Ebola virus can be prevented? | “Stay away from areas where it is prevalent” | AreaAway | 1 |
| Now we’d like you to list all the places where you think you learned any or all of the information you listed about Ebola virus. If you can’t remember where you learned something, describe where you think you would have gone to find this information. | “I've seen reports about it on the nightly news” | NBC Nightly News |  |
| What do you think leads to the common cold? | “Being in close contact with someone who has a cold” | Contact | 1 |
| What do you think makes people more or less likely to develop the common cold? | “Washing hands” | Hand | 1 |
| How do you think the common cold can be prevented? | “Washing hands, not staying in close contact with sick people” | Hand, NoContact | 1  1 |
| Now we’d like you to list all the places where you think you learned any or all of the information you listed about the common cold. If you can’t remember where you learned something, | “fox, cnn, msnbc” | Fox News, CNN News, MSNBC News |  |
| What do you think leads to COVID-19 (the novel coronavirus)? | “Coming in close contact with an infected person” | Contact | 1 |
| What do you think makes people more or less likely to develop COVID-19 (the novel coronavirus)? | Having a weakened immune system, not properly washing hands can make you more susceptible. | Immune Hand | 0  1 |
| How do you think COVID-19 (the novel coronavirus) can be prevented? | “Washing Hands” | Hand | 1 |
| Now we’d like you to list all the places where you think you learned any or all of the information you listed about COVID-19 (the novel coronavirus). If you can’t remember where you learned | “WHO, CDC, Facebook” | Government agency – WHO  Government agency – CDC  Social media - Facebook |  |
| What do you think leads to Zika virus? | “A virus spread through mosquitoes” | Insect | 1 |
| What do you think makes people more or less likely to develop Zika virus? | “People with a weaker immune system” | Immune | 0 |
| How do you think Zika virus can be prevented? | “Can’t be prevented” | Impossible | 0 |
| Now we’d like you to list all the places where you think you learned any or all of the information you listed about Zika virus. If you can’t remember where you learned something, describe where you think you would have gone to find this information. | “In wikipedia during a work search” | Wikipedia | - |
| What do you think leads to food allergies? | “Genetic makeup in your body” | Gene | 1 |
| What do you think makes people more or less likely to develop food allergies? | “Again, most likely their genetics” | Gene | 1 |
| How do you think food allergies can be prevented? | “Cannot be prevented or treated” | Impossible | 0 |
| Now we’d like you to list all the places where you think you learned any or all of the information you listed about food allergies. If you can’t remember where you learned something, describe where you think you would have gone to find this information. | “I have only heard about food allergies from a friend” | Friend |  |
| What do you think leads to Lou Gehrig's disease (ALS)? | “Genetic predisposition to ALS, perhaps?” | Gene | 1 |
| What do you think makes people more or less likely to develop Lou Gehrig's disease (ALS)? | “I honestly don't have any idea” | IDK | - |
| How do you think Lou Gehrig's disease (ALS) can be prevented? | “I do not know if there are any forms of prevention for ALS” | IDK | - |
| Now we’d like you to list all the places where you think you learned any or all of the information you listed about Lou Gehrig's disease (ALS). If you can’t remember where you learned something, describe where you think you would have gone to find this information. | “Google” | Search engine - Google |  |
| What do you think leads to strep throat? | “Bacterial infection leads to strep throat” | Becteria | 1 |
| What do you think makes people more or less likely to develop strep throat? | “Eating healthier with a lot of vitamins” | Diet | 0 |
| How do you think strep throat can be prevented? | “Taking medicine early on that helps sore throats” | Prevent | 0 |
| Now we’d like you to list all the places where you think you learned any or all of the information you listed about strep throat. If you can’t remember where you learned something, describe where you think you would have gone to find this information. | “Family members like my mother growing up, friend who have been infected” | Family  Friend |  |
| What do you think leads to stroke? | “Eating bad things not really normal food” | Diet | 1 |
| What do you think makes people more or less likely to develop stroke? | “Being overweight, having pre-existing conditions such as heart conditions” | Weight  Prexisting | 1  1 |
| How do you think stroke can be prevented? | “eating healthy and staying active” | Diet  Exercise | 1  1 |
| Now we’d like you to list all the places where you think you learned any or all of the information you listed about stroke. If you can’t remember where you learned something, describe where you think you would have gone to find this information. | “Doctor, family members” | Medical professional  Family |  |
