# Supplementary material for: Health Information Sourcing and Health Knowledge Quality: Repeated Cross-sectional Survey
Source: JMIR Form Res. 2022 Sep 28;6(9):e39274. doi: 10.2196/39274 (PMC9557754; doi:10.2196/39274)
Supplement: Multimedia Appendix 6 [file formative_v6i9e39274_app6.docx]

News

Family

Medical professional

Education

Friend

Internet

Social media

Website

Personal experience

WebMD

Search engine

TV

Other people

Government agency

Health organization

Wikipedia

Common/general knowledge

Article

Newspaper

Magazine

Own knowledge

Work

Journal

Movie

Medical materials

Book

Ads

Learning about famous people with disorder

Reading

Educational campaigns

Research

Media

Radio

Documentary

Podcasts

Video

Baseball

Medical studies

Press briefings

Lectures

Airport

Publications

Email

Mail

App

Text alert

Comedian

Video game

Pharmacy

Webinar

Museum

Cartoons

Memes

Religion

Library
