# Supplementary material for: Health Information Sourcing and Health Knowledge Quality: Repeated Cross-sectional Survey
Source: JMIR Form Res. 2022 Sep 28;6(9):e39274. doi: 10.2196/39274 (PMC9557754; doi:10.2196/39274)
Supplement: Multimedia Appendix 7 [file formative_v6i9e39274_app7.docx]

| **Illness** | **Knowledge precision** | **Knowledge depth** |
| --- | --- | --- |
| Ebola | 0.41 | 0.61 |
| Common Cold | 0.49 | 0.51 |
| COVID-19 | 0.76 | 0.72 |
| Zika | 0.43 | 0.68 |
| Food Allergies | 0.33 | 0.42 |
| ALS | 0.24 | 0.27 |
| Strep Throat | 0.51 | 0.53 |
| Stroke | 0.48 | 0.59 |
