# Supplementary figures and images for: Health Information Sourcing and Health Knowledge Quality: Repeated Cross-sectional Survey
Source: JMIR Form Res. 2022 Sep 28;6(9):e39274. doi: 10.2196/39274 (PMC9557754; doi:10.2196/39274)

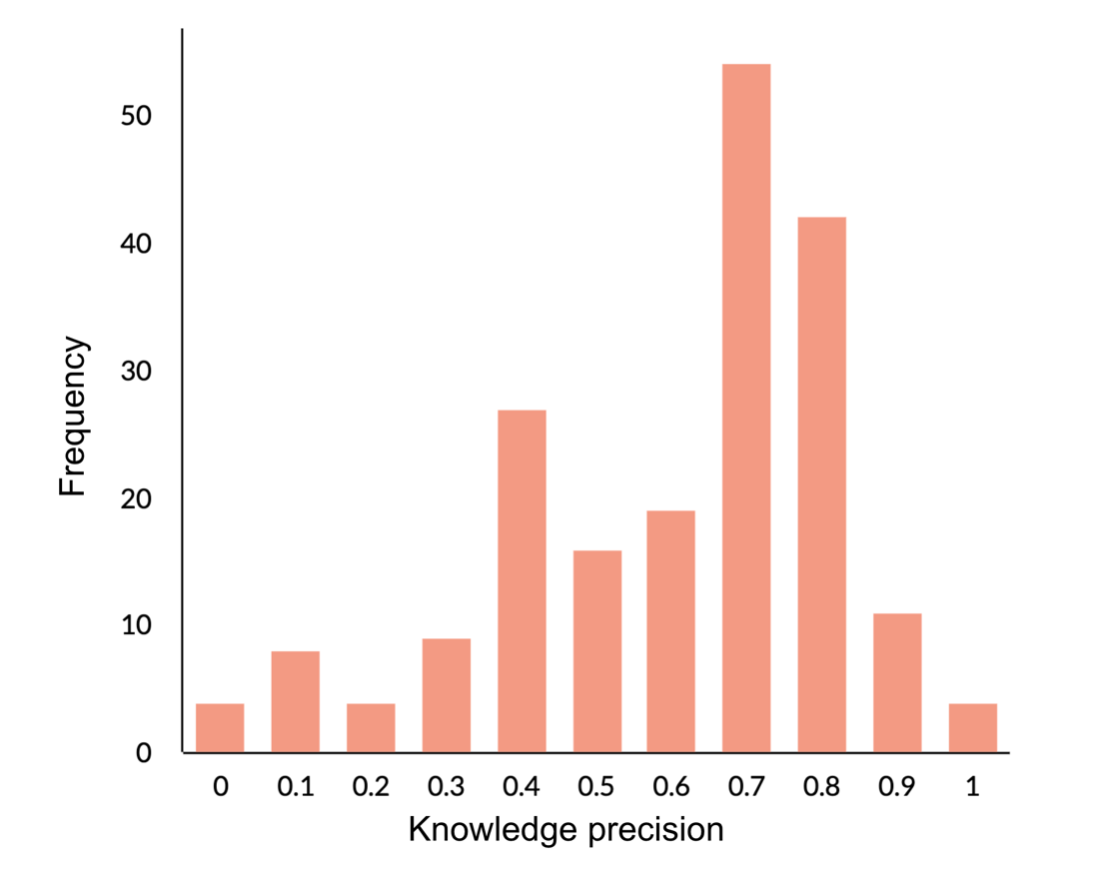

Supplement: Multimedia Appendix 8 [file formative_v6i9e39274_app8.png]

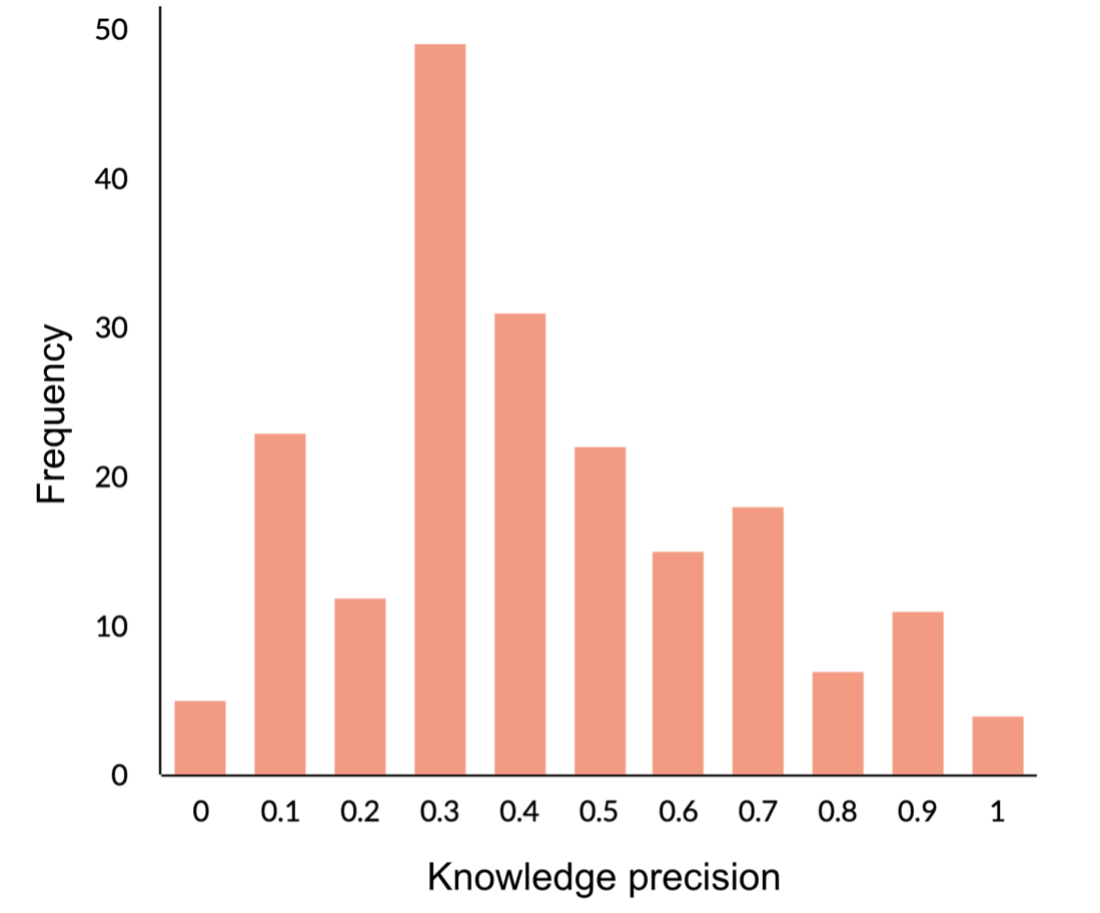

Supplement: Multimedia Appendix 9 [file formative_v6i9e39274_app9.png]

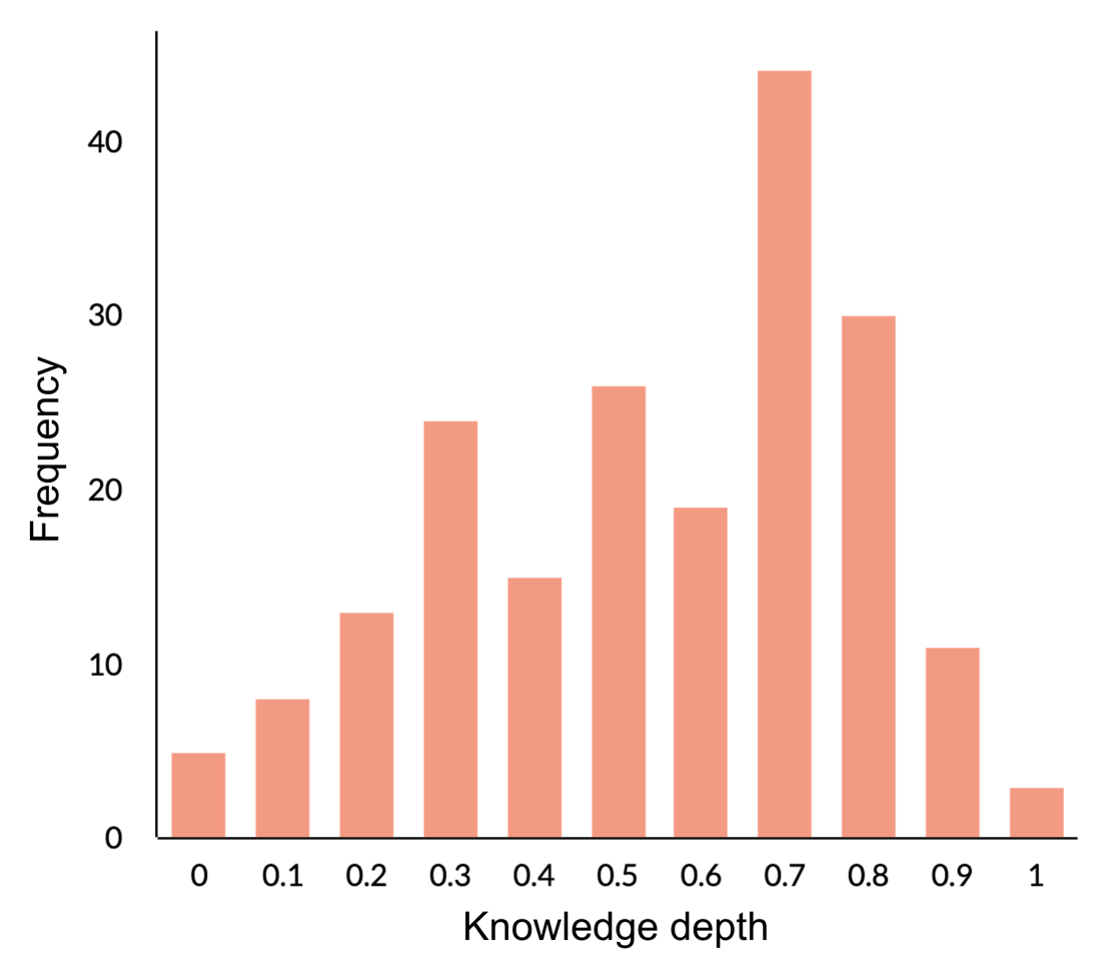

Supplement: Multimedia Appendix 10 [file formative_v6i9e39274_app10.png]

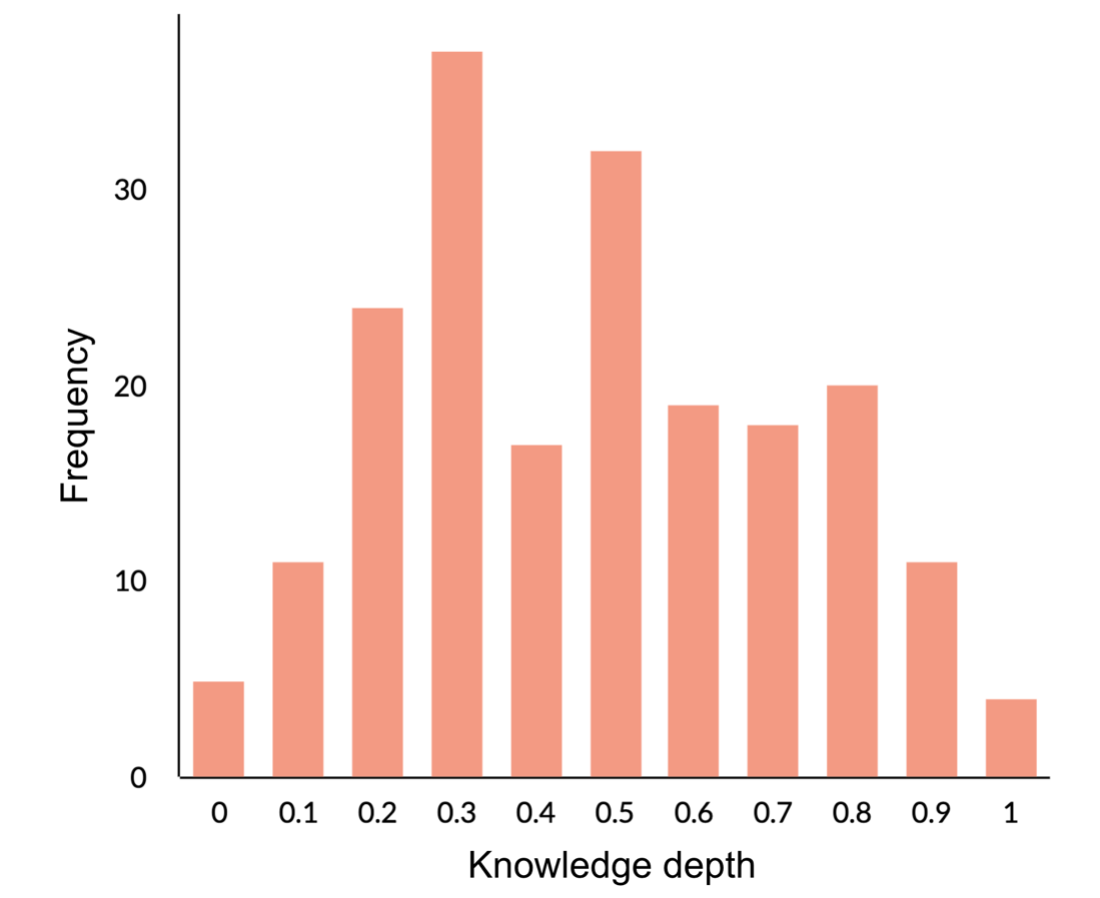

Supplement: Multimedia Appendix 11 [file formative_v6i9e39274_app11.png]
